# Camptothecin Sensitizes Hepatocellular Carcinoma Cells to Sorafenib- Induced Ferroptosis Via Suppression of Nrf2

**DOI:** 10.1007/s10753-023-01823-4

**Published:** 2023-05-12

**Authors:** Ahmed S. Elkateb, Shahira Nofal, Sahar A. Ali, Hanaa B. Atya

**Affiliations:** 1 Biochemistry and Molecular Biology Department, Faculty of Pharmacy, Helwan University, P.O. Box 11795, Cairo, Egypt; 2 Pharmacology and Toxicology Department, Faculty of Pharmacy, Helwan University, P.O. Box 11795, Cairo, Egypt

**Keywords:** sorafenib, ferroptosis, Nrf2 inhibitor, resistance, HCC.

## Abstract

Sorafenib is a potent inducer of ferroptosis used to manage hepatocellular carcinoma (HCC). The ferroptosis induced by sorafenib activates the p62–Keap1–Nrf2 pathway. Abnormal activation of Nrf2 reduces sorafenib’s efficiency and ferroptosis action and induces sorafenib’s resistance. Consequently, our study tried to study the effect of a novel combination of sorafenib and Camptothecin (CPT, Nrf2 inhibitor) to improve sorafenib’s ferroptosis action and reduce sorafenib resistance in the treatment of HCC. We evaluated the efficacy of sorafenib and/or CPT using HepG2 and Huh7 cell lines. MTT assay evaluated the anti-proliferation effects. The combination index (CI) and dose reduction index (DRI) were calculated using Isobologram analysis. Malondialdehyde (MDA), total antioxidant capacity (TAC), iron concentration, glutathione peroxidase (GPX4), and glutathione reductase (GR) activity assays were used to determine the ferroptosis action of drugs. Western blot was used to investigate the expression of the implicated proteins. Bioinformatics tools were used to determine the correlation between these proteins. Finally, the HPLC technique is used to measure cellular drug uptake. Our results revealed a strong synergism between sorafenib and CPT. The synergetic combination significantly increases lipid peroxidation and iron concentration, decreases TAC, GPX4 and GR activity, and reduces the expression of both Nrf2 and SLC7A11. The downregulation of Nrf2 expression has a vital role in the reduction of resistance mediators to sorafenib against HCC cells like (p62, MT1G, and ABCG2) and improves the cellular uptake of sorafenib. The current study provided evidence that Nrf2 inhibition by CPT improves sorafenib’s sensitivity and reduces sorafenib’s resistance via the augmentation of sorafenib’s ferroptosis action.

## INTRODUCTION

Hepatocellular carcinoma (HCC) is the world's sixth most prevalent cancer and the third leading cause of cancer mortality [1]. 80% of HCC patients are diagnosed at an advanced stage of the disease, with only a 6–8-month median survival [2]. Therefore, chemotherapeutic treatment becomes the most used intervention strategy and one of the few options available [3].

In patients with advanced HCC, sorafenib has been found to increase survival. Because of this, the Food and Drug Administration (FDA) has authorized it as a first-line therapy for advanced HCC [4]. Yet, several patients had acquired resistance and adverse effects, including skin toxicity and arterial hypertension [5]. Sorafenib is a multi-target kinase inhibitor and ferroptosis inducer via inhibiting the activity of cystine/glutamate transporter (SLC7A11/x CT) [6]. Ferroptosis is a defined kind of programmed cell death characterized by increasing reactive oxygen species (ROS) production and lipid peroxide accumulation to lethal levels [7].

Under ferroptosis's oxidative stress, cancer cells stimulate p62 (also known as SQSTM1) which promotes autophagic degradation of Kelch-like ECH-associated protein 1 (Keap1), resulting in a positive feedback loop of continuous nuclear factor E2-related factor 2 (Nrf2) activation to maintain oxidative homeostasis [8, 9]. The activation of Nrf2 results in the upregulation of some genes involved in the prevention of lipid peroxidation and ferroptosis such as glutathione reductase (GR), and SLC7A11 [10, 11].

Additionally, sorafenib activates directly the p62-Keap1-Nrf2 antioxidative signaling pathway, accordingly, sorafenib's ability to induce ferroptosis was consequently reduced [6]. Growing evidence suggests that overexpression of Nrf2 promotes the expression of p62, Metallothionein -1G (MT-1G), and ATP binding box G2 (ABCG2) which participate in acquired sorafenib resistance [12–14]. As a result, cancer cells may develop sorafenib resistance and protect themselves from ferroptosis by overexpression of Nrf2 [15].

Camptothecin (CPT), a repurposing topoisomerase inhibitor, is one of the most potent Nrf2 inhibitors through reducing transcription, translation, and/or increasing mRNA degradation of Nrf2 [3, 16, 17]. But until now the CPT's role in regulating ferroptosis, reducing the resistance of sorafenib and improving antitumor activity in HCC cells remains unknown. In addition, the relation between ferroptosis, sorafenib resistance mechanisms and Nrf2 expression is still unclear. Consequently, the purpose of our research was to shed light on studying the combined effect of sorafenib and Camptothecin (Nrf2 inhibitor) in improving sorafenib’s ferroptosis action and reducing sorafenib resistance by investigating various resistance pathways implicated in HCC.

## MATERIALS AND METHODS

### Materials

#### Drugs and Reagents

Sorafenib, Camptothecin, and Dimethyl fumarate (DMF, Nrf2 inducer) were purchased from (Tocris Biosciences). Ferrostatin-1 and ZVAD-FMK were obtained from Sigma-Aldrich (Shanghai, China). Dimethyl sulfoxide (DMSO) and 3-(4,5-dimethyl thiazolyl-2)-2,5-diphenyltetrazolium bromide (MTT) were provided from (Sigma-Aldrich, USA, and Serva^®^ Germany respectively). Dulbecco's Modified Eagle's Medium (DMEM), penicillin, streptomycin, fetal bovine serum, and phosphate buffer saline (PBS) were obtained from (Gibco-USA). RIPA buffer (50 mM Tris–HCl (pH 7.4), 150 mM NaCl, 5 mM EDTA, 1% Triton X-100, 0.5% Sodium deoxycholate, and 0.1% SDS), HEPES buffer (4-(2-hydroxyethyl)-1-piperazineethanesulfonic acid) were purchased from (Biowest, India). Antibodies against Nrf2, p62, SLC7A11, MT-1G, and ABCG2 were provided by (Abcam, UK).

#### Cell Lines

Hepatocellular carcinoma (HepG2 and Huh7) cell lines were purchased from (Nawah Scientific^®^, Egypt). Cell lines were grown in Dulbecco's Modified Eagle's Medium (DMEM) supplemented with penicillin (100 U/ml), streptomycin (100 mg/ml), and 10% fetal bovine serum (Gibco-USA).

### Methods

Cell lines were maintained in the Center of Excellence "Helwan Structural Biology Research, HSBR" under standard laboratory conditions (10% inactivated fetal bovine serum (FBS) and 1.0% penicillin–streptomycin and kept at 37 ^o^ C with 5% CO_2_). Drug stock solutions were prepared in DMSO. All experiments were repeated three times.

#### Cell Viability Assay

The viability of studied cell lines after treatment with (sorafenib or CPT) and different combinations of two drugs with a constant concentration ratio (1:1) were measured using a colorimetric MTT assay as described by van Meerloo et al. [18]. Briefly, in 96-well plates, cells were cultured at a density of 7500 cells per well, and the plates were then left to adhere for the night. Then, five series concentrations (0.01, 0.1, 1, 10, and 100 μM) of sorafenib [19], CPT, and various combinations of the two drugs at constant concentration ratios (1:1) were added to the wells. These concentrations were prepared by diluting the stock solutions with DMEM. Cells treated with 0.1% DMSO were used as a negative control. For each cell line, two plates were prepared one for 48 h and the other for 72 h and were maintained under standard culture growth conditions. Following the incubation time, 20 μl MTT (5 mg/ml in PBS) is added and incubated for 4 hrs. Following this, 150 μl DMSO is added to each well to dissolve the crystals that had formed. The absorbance of solubilized violet crystals was measured at 540 nm using Biotek 800 TS microplate reader (Vermont, USA). The five concentrations of each drug and combination were plotted versus the calculated cell viability using GraphPad Prism 7 and the IC_50_ was interpolated using the 4-parameter logistic model (4PL).

In addition, we used MTT assay to investigate the mechanism of cell death induced by the used combination (sorafenib and CPT). So, we treated both cell lines with another two compounds. The first, the cells treated with 1 μM of ferrostatin-1 (a ferroptosis inhibitor) to the combination of sorafenib and CPT, and the second treated with 10 μM of ZVAD-FMK (an apoptosis inhibitor) to the combination of sorafenib and CPT [20].

#### Isobolgram Analysis

Based on the isobologram technique established by Chou and Talalay [21]. The multiple-drug effect analysis between sorafenib and CPT on both cell lines after 48 and 72 hrs. of incubation using CompuSyn software (Version1, Cambridge, United Kingdom) was done.

The MTT results were used to calculate the combination index (CI) where CI < 1 indicates synergism, CI = 1 indicates additive or CI > 1 indicates antagonism. Furthermore, the software determines the dose reduction index (DRI) which estimates a fold-decrease in the dose of each drug when compared to their combined dose [22]. Depending on CI and DRI results, we selected the best time of incubation for further investigations.

#### Cell Lysate Preparation

Cells were seeded at a density of 1 × 10^6^ cells per flask (15 flasks for each cell line) and were allowed to adhere overnight. Then, flasks were categorized into five groups; the first group was a control (1% DMSO), the second group was treated with IC_50_ of sorafenib, the third group was treated with IC_50_ of CPT, and the fourth group was treated with a mixture of CPT and sorafenib at their lowered synergistic doses determined by DRI values, and the last group was treated with IC_50_ of sorafenib and DMF (at their IC_50_ values, (IC_50_ of DMF = 100 μM) [23].

All groups were incubated for 72 hrs. Subsequently, cells were lysed in pre-cold RIPA lysis buffer supplemented with protease and phosphatase inhibitors cocktail (Sigma-Aldrich) as described by El-Hanbosy et al. [24] and incubated for 30 min on ice. Then cells were scraped, vortexed, and centrifuged at 13,000 rpm for 15 min. The Bradford method was used to calculate the amount of total proteins in the supernatant [25]. Finally, cell lysate was aliquoted and stored at -80 °C until they were used in the following investigations.

#### Ferroptosis Markers

- A. Total Antioxidant Capacity

The total antioxidant capacity (TAC) in the cell lysate of different HCC cell lines was evaluated calorimetrically according to Koracevic et al. [26] using (Biodiagnostics^®^ kit, Cairo, Egypt) according to the manufacturer’s protocol. Then, TAC in each sample was calculated using the following equation.

$${^{\prime\prime}}TAC\;(mM)=(A\;blank-A\;sample)\;\ast\;3.33\,{^{\prime\prime}}$$

- B. Lipid Peroxide (Measured as Malondialdehyde (MDA))

The malondialdehyde (MDA) content in cell lysate was measured using (Biodiagnostics^®^ kit, Cairo, Egypt) according to the manufacturer’s protocol. The MDA concentration in each sample was calculated using the following equation.

$${^{\prime\prime}}\!MDA\;(nmol/ml)=A_{sample}\;/A_{standard}\;\ast\;{10}\,{^{\prime\prime}}$$

- C. Glutathione Reductase (GR) Activity

The Glutathione reductase (GR) activity was reported by the kinetic method according to Illingworth J using (Biodiagnostics kits, Egypt) according to the manufacturer’s protocol [27]. Then, the absorbance was measured simultaneously every minute for 5 min, and the change in absorbance per min was measured. Finally, the glutathione reductase activity in each sample was calculated using the following equation.

$$Enzyme\;Activity\,(U/ml)=\frac{(\Delta\;A\;{340}\;nm\;/min)\;\ast\;Total\;volume\;(in\;milliliters)\;of\;assay}{6.{22}\;\ast\;volume\;of\;sample}$$

6.22 = Millimolar extinction coefficient of ß-NADPH at 340 nm

- D. Glutathione Peroxidase (GPX4)

The Glutathione Peroxidase (GPX4) activity was measured by kinetic method according to the manufacturer’s protocol (Biodiagnostics^®^ kit, Cairo, Egypt). Then, the absorbance was measured simultaneously every minute for 3 min, and the change in absorbance per min was measured. Finally, the glutathione peroxidase (GPX4) activity in each sample was calculated using the following equation.

$$Enzyme\;Activity\,(U/ml)=\frac{{(\triangle\;A\;{340}\;nm/min)}\;\ast \;Total\;volume\;(in\;milliliters)\;of\;assay}{6.{22}\;\ast\; volume\;of\;sample}$$

6.22 = Millimolar extinction coefficient of ß-NADPH at 340 nm

- E. Iron Assay

The relative iron concentration in cell lysates was measured using (Biodiagnostics^®^ kit, Cairo, Egypt) according to the manufacturer’s protocol. The iron concentration in each sample was calculated using the following equation.

$${^{\prime\prime}}\mathrm{Iron}\;\mathrm{concentration}\;(\mu\mathrm{mol}/\mathrm L)=({\mathrm A}_{\mathrm{sample}}\;/{\mathrm A}_{\mathrm{Standard}})\;\ast\;35.8{^{\prime\prime}}$$

35.8 = Concentration of standard

#### Cellular Uptake Quantitative Analysis

To form cell monolayers, 2 × 10^5^ cells/mL was seeded per well into six-well plastic plate for each cell line. The cells were then rinsed with 200 µl of HBSS (uptake buffer) and allowed to equilibrate for 1 h in an incubator after reaching 80 to 90% confluence. Upon the removal of the HBSS, for each cell line, plates were categorized into three groups: The first group treated with IC_50_ of sorafenib, a second group treated with the IC_50_ of CPT and the third one treated with mixture of CPT and sorafenib at their lowered synergistic doses determined by DRI values and were incubated for 3 hrs. Then, the cells were harvested with a spatula. The cell lysate was transferred to an Eppendorf tube and centrifuged at 15,000 rpm for 10 min after being washed four times with 4 °C PBS. High-performance liquid chromatography (HPLC) (LC-20AT; Shimadzu, Kyoto, Japan) equipped with an SPD-20 A UV/VIS detector was used to quantitatively evaluate the amounts of sorafenib and CPT in the cells. The mobile phase was 0.1% Trifluoro acetic acid: Acetonitrile gradient elution with a flow rate: 1 ml/min; column temperature, 25 °C; UV detection wavelength 260 nm; and injection volume, 50 µl. The samples were extracted using acetonitrile [28]. The data were calculated by measuring drug quantity in cells compared to the initial drug amount in media %.

#### Western Blotting Analysis

The expression of Nrf2, p62, SLC7A11, MT-1G, and ABCG2 proteins in Huh7 and HepG2 cells treated with sorafenib alone, CPT alone and a combination of CPT and sorafenib at their reduced synergistic doses were determined using western blot analysis according to the method described by Burnette WN [29].

#### Statical Analysis

All data were expressed as the mean ± SEM. The differences between the groups were analyzed using unpaired student t- test and one-way analysis of variance (ANOVA) followed by post hoc Tukey's multiple comparison test and graphical data were represented using Graph Pad Prism^®^ 7 (GraphPad Software Inc., CA, USA). Isobologram analysis was performed by CompuSyn software (Version1, Cambridge, United Kingdom). Western blot bands were analyzed by Total lab analysis software (Ver.1.0.1). Empower software (Version 3) was used for HPLC analysis. STRING database tool was used to predict protein–protein interactions and identify the correlation between proteins [30].Statistical differences were significant when p < 0.05.

## RESULTS

### Cytotoxicity Effects of the Studied Drugs

**Fig. 1:**
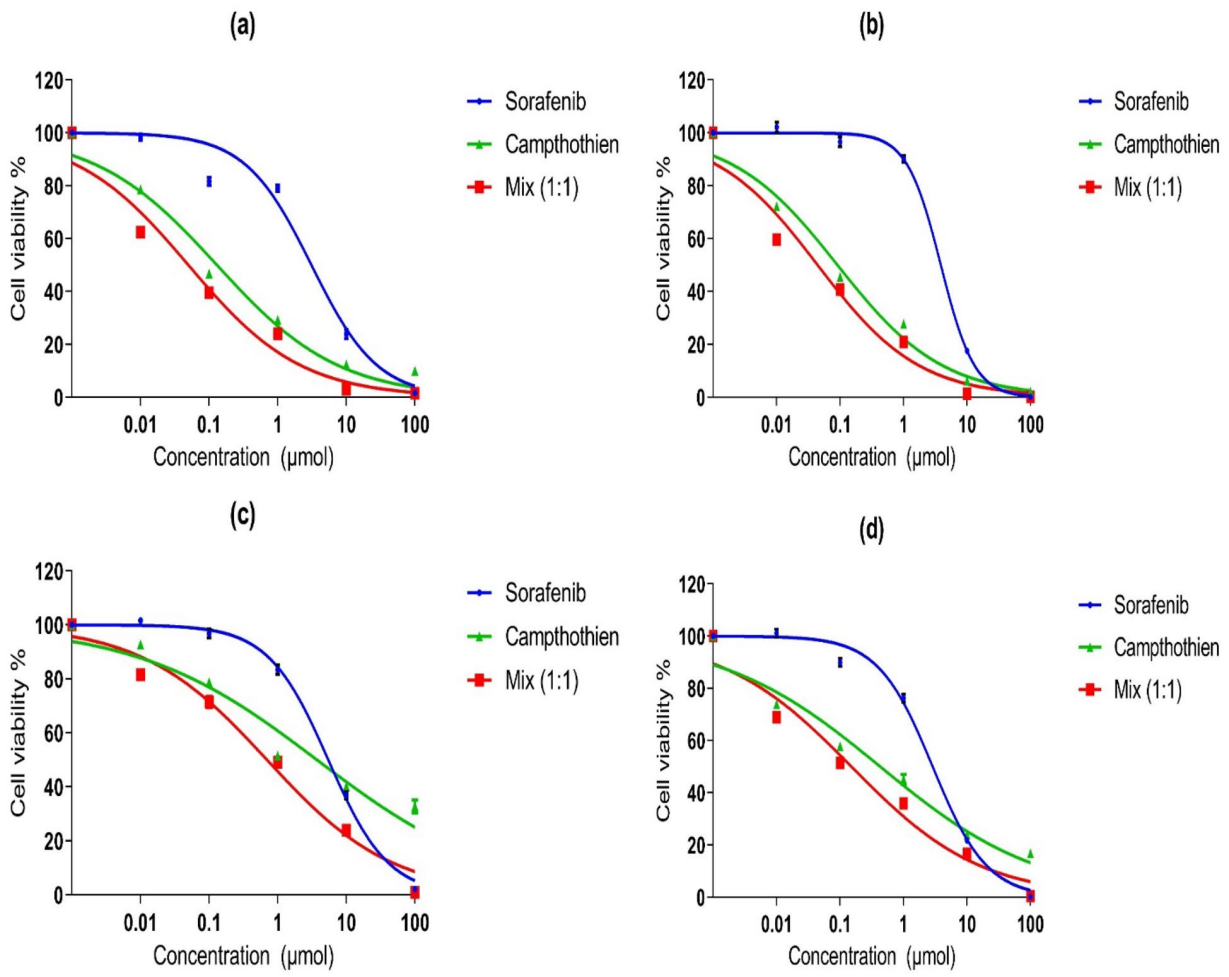
The Cytotoxicity effect of sorafenib, Camptothecin (CPT) and Mixture of sorafenib and CPT with a ratio (1: 1) in HepG2 and Huh7 cell lines in two different incubation periods (48 and 72 hrs.) determined by the MTT assay **a** Dose–response curves of drugs in HepG2 cells after 48 hrs. of incubation. **b** Dose–response curves of drugs in HepG2 cells after 72 hrs. of incubation. **c** Dose–response curves of drugs in Huh7 cells after 48 hrs. of incubation **d** Dose–response curves of drugs in Huh7 cells after 72 hrs. of incubation. Data are presented as mean ± SEM of at least three independent times.

In HepG2 cell line, sorafenib and CPT had IC_50_ values (3.1 ± 0.49 µM and 0.13 ± 0.02 µM, respectively) after 48 hrs. of incubation. Additionally, the combination of sorafenib and CPT with ratio (1:1) inhibited cell viability with an IC_50_ equal to 0.05 ± 0.01 µM as shown in Fig. 1a. After 72 hrs. of incubation, sorafenib showed an IC_50_ equal to 3.87 ± 0.21 µM but CPT’s IC_50_ was 0.091 ± 0.01 µM. While the IC_50_ for the combination of sorafenib and CPT was 0.044 ± 0.01 µM as shown in Fig.1b.

In Huh7 cell line after 48 hrs. of incubation, sorafenib showed an IC_50_ (5.47 ± 0.31 µM), while CPT showed an IC_50_ (1.04 ± 0.93 µM). The combination showed an IC_50_ (0.33 ± 0.12 µM) as shown in Fig. 1c.

But after 72 hrs. of incubation, sorafenib and CPT had IC_50_ values (2.88 ± 0.24 and 0.4 ± 0.09 µM, respectively). While a combination of sorafenib and CPT showed an IC_50_ equal to 0.152 ± 0.03 µM as shown in Fig. 1d.

The results demonstrated that the combination of sorafenib and CPT significantly augmented cell growth inhibition more than each drug alone. The combination is more potent after 72 hrs. than 48 hrs. of incubation.

### Isobologram Analysis for a Combination of Sorafenib and Camptothecin (CPT)

**Fig. 2:**
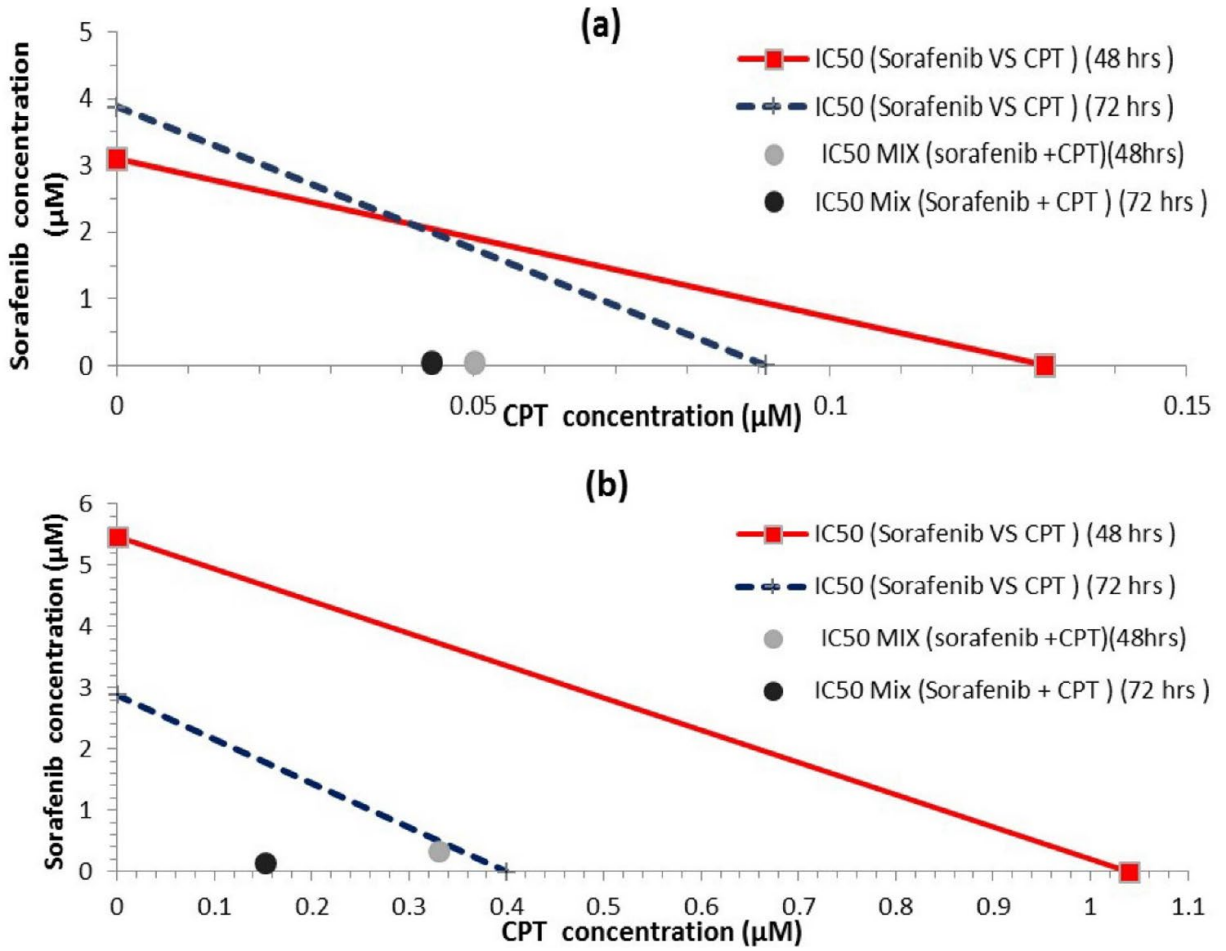
Isobologram analysis of **a** HepG2 cells after 48 and 72 hrs. of exposure to sorafenib in combination with CPT **b** Huh7 cells after 48 and 72 hrs. of exposure to sorafenib in combination with CPT. The zones of antagonistic (upper), synergistic (lower), and additivity (on the line) are illustrated. The data point’s distance from its respective line indicates the degree of synergism in this drug combination (same color). The results show that there is synergism at the IC_50_ level. The data points in circles are the means of three different replicates.

Combination index (CI) and DRI at the IC_50_ dose obtained from MTT assay were calculated using CompuSyn software to determine the type of drug interaction and the results expressed in Table 1 and Fig. 2.

**Table 1:** Combination index (CI) and Dose reduction index (DRI) of each drug calculated via CompuSyn software at IC_50_ upon treating HepG2 and Huh-7 cell lines for 48 and 72 h

**Cell line**	**Time of incubation**	**Drugs**	**CI Value at IC**_**50**_	**IC**_**50**_** of each drug alone (µM)**	**IC**_**50**_** of each drug in combination (µM)**	**DRI**
**HepG2**	**48 hrs.**	**sorafenib**	0.398	3.11 ± 0.49	0.05 ± 0.02	62.4
		**CPT**		0.13 ± 0.02		2.612
	**72 hrs.**	**sorafenib**	0.494	3.87 ± 0.21	0.044 ± 0.01	87.95
		**CPT**		0.091 ± 0.01		2.068
**Huh7**	**48 hrs.**	**sorafenib**	0.377	5.47 ± 0.31	0.33 ± 0.12	16.57
		**CPT**		1.04 ± 0.93		3.15
	**72 hrs.**	**sorafenib**	0.435	2.88 ± 0.2	0.152 ± 0.03	18.87
		**CPT**		0.4 ± 0.09		2.616

IC_50_ values were determined from the MTT *in-vitro* antiproliferation assay by non-linear regression of dose–response curves using the 4-parameter logistic model (4PL) in GraphPad prism 7. Data represent mean ± SEM, n = 3

At IC_50_ after 48 hrs. of incubation, CI value was (0.398) in HepG2 and (0.377) in Huh7 cell lines. These CI values, which are markedly lower than 1, indicate a synergism in sorafenib and CPT combination in both Huh7 and HepG2 cell lines.

Furthermore, the same scenario occurred after 72 hrs. of incubation, CI was (0.494) in HepG2 and (0.435) in Huh7cells revealing a synergistic effect for sorafenib and CPT combination in both Huh7 and HepG2 cell lines.

In addition, the DRIs exhibited remarkable dose reduction for sorafenib and CPT where, the IC_50_ of sorafenib decreased by (62.24-fold and 16.57-fold in both HepG2 and Huh7 cell lines, respectively) after 48 hrs. of incubation. While after 72 hrs. of incubation, the IC_50_ of sorafenib decreased by (87.95 -fold and 18.87-fold in both HepG2 and Huh7 cell lines, respectively). The DRIs results clearly indicated that addition of CPT to sorafenib potentiates its efficacy.

The results of the MTT, CI, and DRI clearly showed that the synergetic combination of sorafenib and CPT after 72 hrs. of incubation is more effective than 48 hrs. of incubation.

CI < 1, CI = 1, or CI > 1, indicate synergistic effect, additive effect, or antagonistic effect, respectively. DRI was calculated by comparing doses when used as a single treatment or in combination. CI stands for combination index, and DRI stands for dose reduction index.

**Fig. 3:**
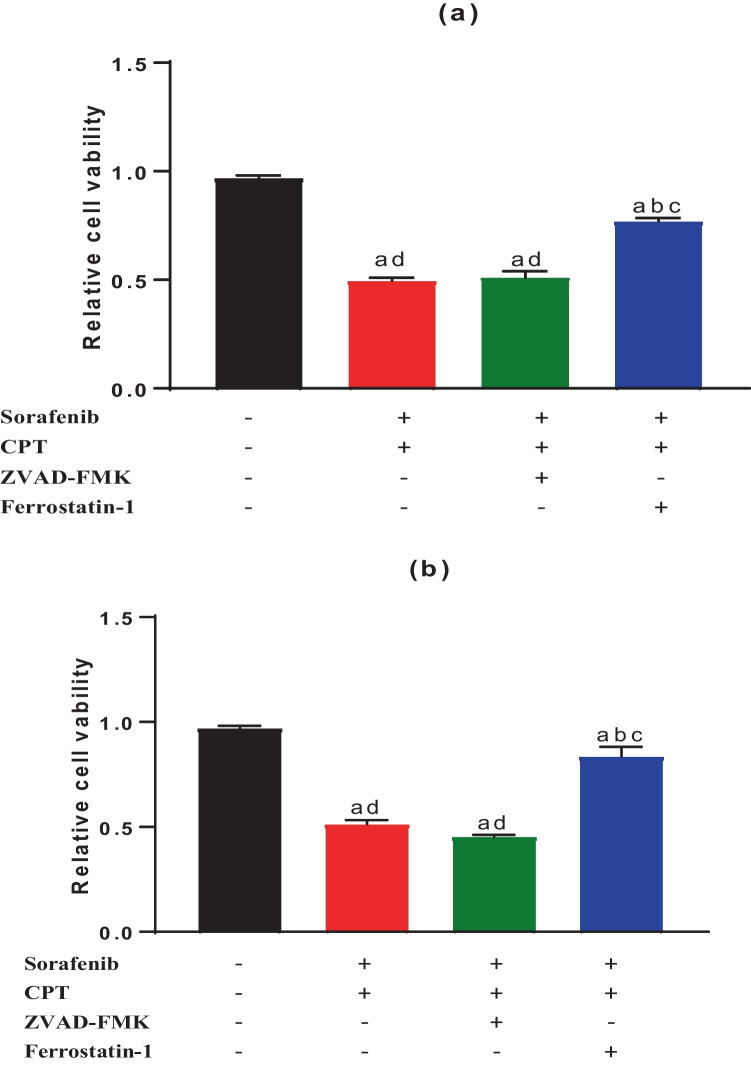
The cell viability in HepG2 cell line **a** and Huh7 cell line **b** after treatment with (sorafenib and CPT), (sorafenib and CPT + ferrostatin-1), and (sorafenib and CPT + ZVAD-FMK) for 72 hrs. Data are presented as Mean ± SEM, n = 3/group. a: significant difference from the control group, b: significant difference from mix 1 (sorafenib and CPT) group, c: significant difference from (sorafenib, CPT, and ZVAD-FMK) and d: significant difference from (sorafenib, CPT and ferrostatin-1) group, using one-way ANOVA followed by Tukey- Kramer multiple comparisons test.

In addition, in both cell lines, the cells treated with a combination of (sorafenib, CPT and ferrostatin-1) showed a significant increase in cell viability compared to both cells treated with a combination of (sorafenib, CPT and ZVAD-FMK) and cells treated with a combination of sorafenib and CPT alone *(p* < 0.0001, *p* < 0.0001, respectively), and decreased significantly compared to control group (*p* < 0.0001) as shown in Fig. 3a, b.

Furthermore, cells treated with a combination of (sorafenib, CPT and ZVAD-FMK) did not show any significant difference in cell viability compared to cells treated with a combination of sorafenib and CPT alone. So, cell viability was restored by ferrostatin-1 while not by ZVAD-FMK in both cell lines (Fig. 3a, b). These findings indicate that the combination of sorafenib and CPT suppressed HCC cell proliferation via ferroptosis.

### Effect of Combination on Ferroptosis Markers

#### Total Antioxidant Capacity (TAC)

**Fig. 4:**
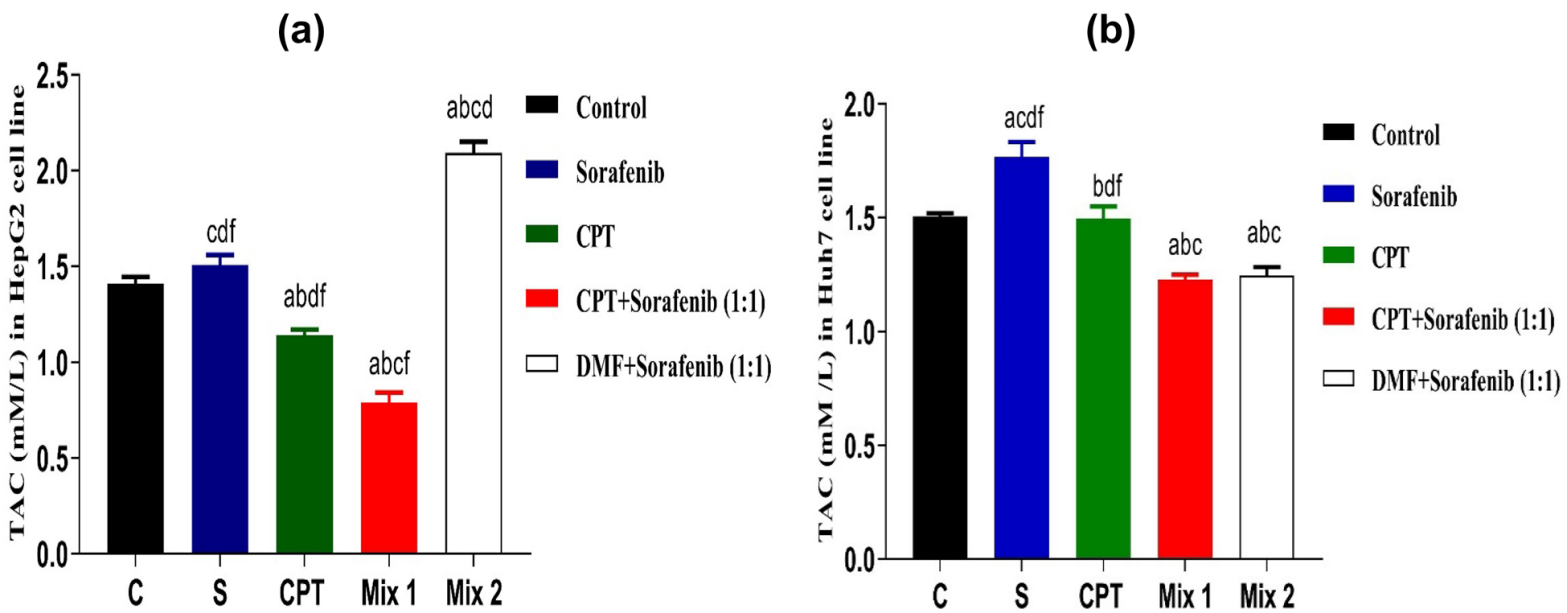
Total antioxidant capacity (TAC) in HepG2 cell line **a** and Huh7 cell line **b** after treatment with IC_50_ of the sorafenib, CPT, mix1 (combination of CPT and sorafenib) and mix 2 (Sorafenib and Dimethyl fumarate) for 72 hrs. Data are presented as Mean ± SEM, n = 3/group. a: significant difference from the control group, b: significant difference from sorafenib, c: significant difference from CPT group, d: significant difference from mix1 group, and f: significant difference from mix2 group, using one-way ANOVA followed by Tukey- Kramer multiple comparisons test.

Total antioxidant capacity (TAC) was determined calorimetrically in HepG2 and Huh7 cell lysates in 72 hrs. The results showed that in HepG2 cell line, TAC was decreased significantly by using mix 1 (combination of CPT and sorafenib) (0.7914 ± 0.05 mM/L) compared to both sorafenib group (1.504 ± 0.05 mM/L, *p* < 0.0001), CPT group (1.14 ± 0.03 mM/L, *p* < 0.01), control group (1.407 ± 0.037 mM/L,* p* < 0.0001) and mix 2 (combination of sorafenib and Dimethyl fumarate; Nrf2 inducer) (2.09 ± 0.05 mM/L,* p* < 0.0001). While mix 2 showed statistically significant increase in TAC value when compared to mix 1, sorafenib, CPT and control (*p* < 0.0001, *p* < 0.0001,* p* < 0.0001,* p* < 0.0001 respectively) as shown in Fig. 4a.

Furthermore, the same scenario occurred in Huh7 cell line, mix 1 showed a significant decrease in TAC value (1.228 ± 0.02 mM/L) compared to both sorafenib group (1.766 ± 0.067 mM/L, *p* < 0.0001), CPT group (1.496 ± 0.05 mM/L,* p* < 0.01) as well as control group (1.5 ± 0.01 mM/L,* p* < 0.01) but it did not show significant differences compared to mix 2 group. While mix 2 showed statistically significant increase in TAC value when compared to mix1, CPT and control (p < 0.0001, *p* < 0.001,* p* < 0.001 respectively) as shown in Fig. 4b.

According to these findings, adding CPT to sorafenib decreases the total antioxidant capacity (TAC), hence increasing ROS level, and inducing ferroptosis action in both cell lines.

#### Lipid Peroxide (Measured as Malondialdehyde (MDA)

**Fig. 5:**
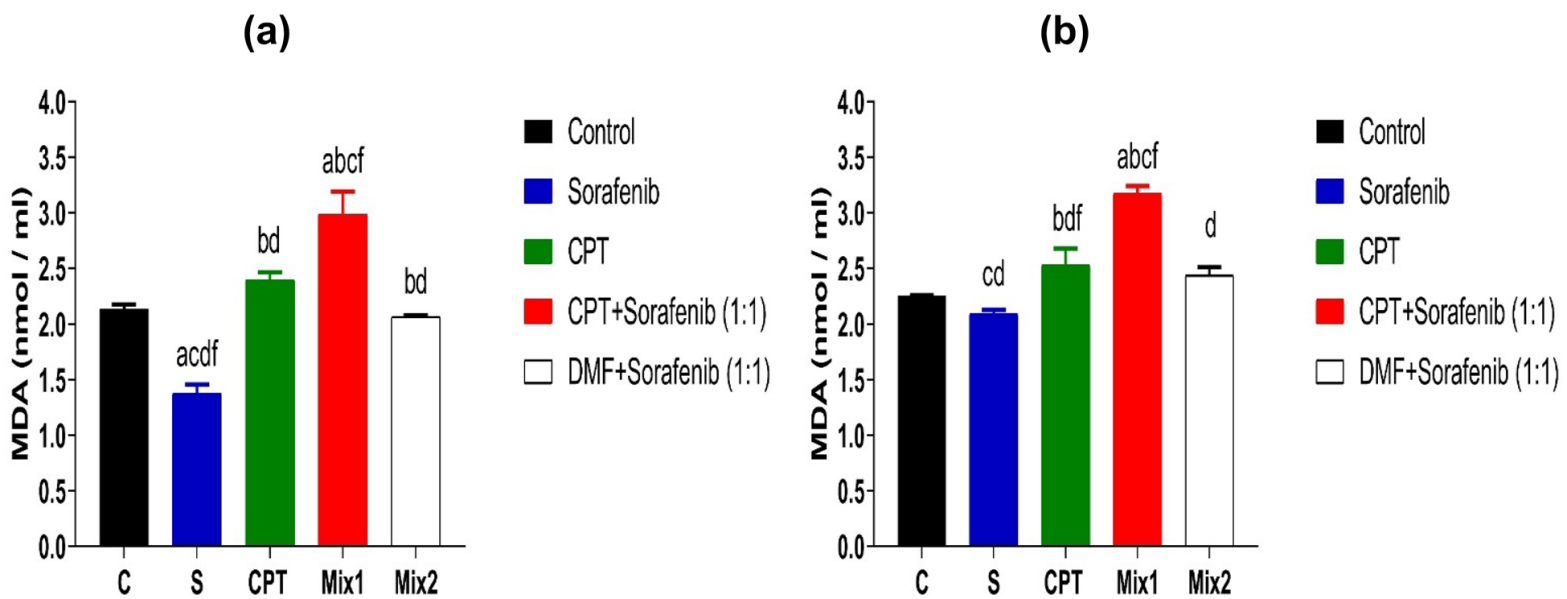
Malondialdehyde levels (MDA) in HepG2 **a** and Huh7 cells **b** after 72 hrs. of treatment with IC_50_ of Sorafenib, CPT, mix 1 (combination of CPT and sorafenib), and mix 2 (sorafenib and Dimethyl fumarate). Data are presented as Mean ± SEM, n = 3/group. a: significant difference from the control group, b: significant difference from sorafenib, c: significant difference from CPT group, d: significant difference from mix1 group, and f: significant difference from mix2 group, using one-way ANOVA followed by Tukey- Kramer multiple comparisons test.

In HepG2 cell line, mix 1 showed a significant increase in MDA level (2.982 ± 0.2 nmol/ml) compared to sorafenib (1.374 ± 0.08 nmol/ml, *p* < 0.0001), CPT (2.396 ± 0.069 nmol/ml,* p* < 0.05) mix2 (2.069 ± 0.01 nmol/ml,* p* < 0.001) and control group (2.135 ± 0.04 nmol / ml,* p* < 0.01). Furthermore, MDA value was significantly decreased by using Mix2 compared to control, mix1 and CPT (*p* < 0.01, *p* < 0.0001, *p* < 0.001, respectively) as shown in Fig. 5a.

While in Huh7 cell lysate, MDA level increased significantly in the mix1 (3.17 ± 0.07 nmol/ml) compared to the sorafenib group (2.091 ± 0.039 nmol/ml, *p* < 0.0001), CPT group (2.527 ± 0.15 nmol/ml,* p* < 0.01), mix2 (2.43 ± 0.08 nmol/ml,* p* < 0.001) and control group (2.25 ± 0.01 nmol / ml,* p* < 0.001) as shown in Fig. 5b. Additionally, mix 2 showed statistically significant decrease in MDA value when compared to mix1 (*p* < 0.001). This may indicate that the combination of CPT with sorafenib causes an increase in lipid peroxide in both cell lines and accelerates cell death via ferroptosis.

#### Glutathione Reductase (GR)

**Fig. 6:**
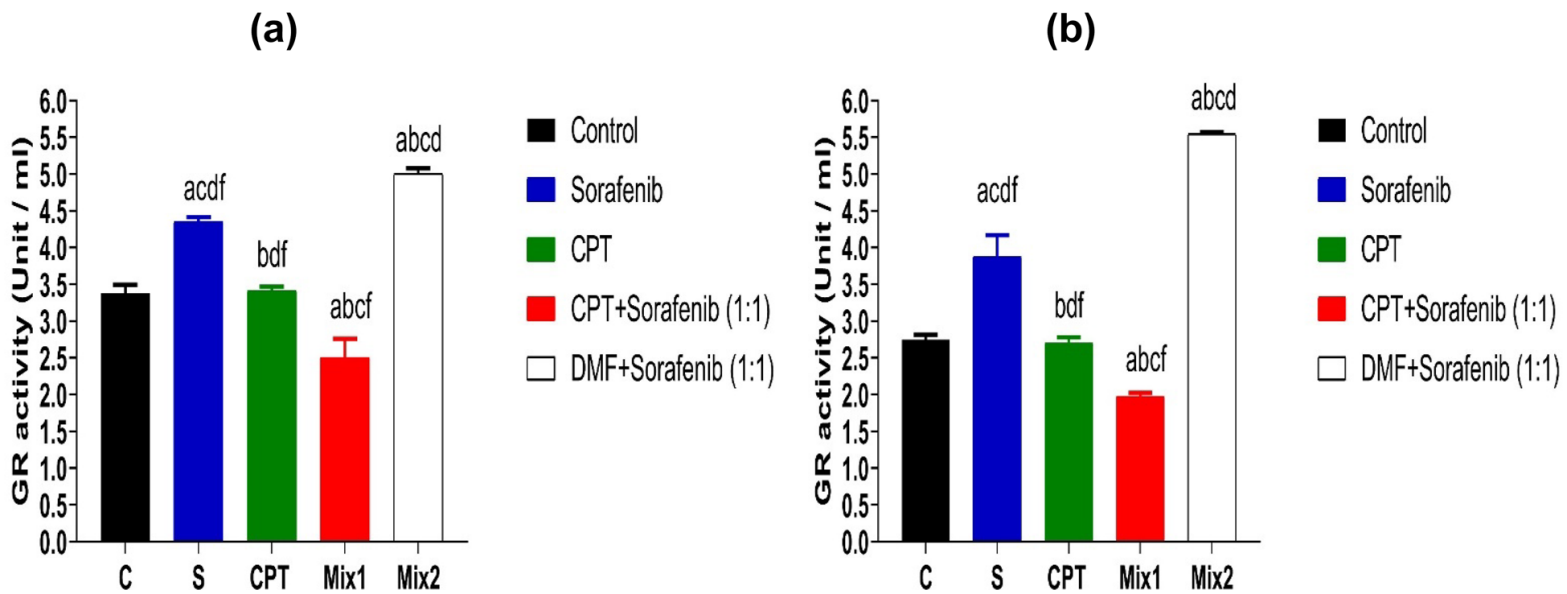
Glutathione reductase (GR) activity in HepG2 **a** and Huh7 cells **b** after 72 hrs. of treatment with IC_50_ concentrations of sorafenib, CPT, mix 1 (combination of CPT and sorafenib), and mix 2 (sorafenib and Dimethyl fumarate). Data are presented as Mean ± SEM, n = 3/group. a: significant difference from control group, b: significant difference from sorafenib, c: significant difference from CPT group, d: significant difference from mix1 group, and f: significant difference from mix2 group, using one-way ANOVA followed by Tukey- Kramer multiple comparisons test.

In HepG2 cells, GR activity showed a significant decrease in cells treated with mix 1 (2.503 ± 0.3 U/ml) compared to both sorafenib (4.349 ± 0.07 U/ml*, p* < 0.0001), CPT (3.415 ± 0.06 U/ml, *p* < 0.01), mix 2 (5.008 ± 0.076, *p* < 0.0001) and control groups (3.383 ± 0.11 U/ml, *p* < 0.01) as shown in Fig. 6a. While mix 2 showed a statistically significant increase in GR activity when compared to mix1, CPT, control and sorafenib (*p* < 0.0001, *p* < 0.0001,* p* < 0.0001,* p* < 0.05 respectively).

Also, the same results occurred in Huh7 cell lysate, by using mix 1, the GR activity decreased significantly (1.973 ± 0.05 U/ml) compared to sorafenib (3.383 ± 0.11 U/ml, *p* < 0.0001), CPT (2.931 ± 0.09 U/ml, *p* < 0.01), mix 2 (5.549 ± 0.025 U/ml, *p* < 0.0001), and control groups (2.747 ± 0.07 U/ml, *p* < 0.01) as shown in Fig. 6b. Furthermore, mix 2 showed statistically significant increase in GR activity when compared to mix1, CPT, control and sorafenib (*p* < 0.0001, *p* < 0.0001,* p* < 0.0001,* p* < 0.0001 respectively).

These findings demonstrated that GR activity in both cell lines was declined after the addition of CPT to sorafenib. So, sorafenib's ferroptosis activity may be improved by Nrf2 suppression caused by CPT.

#### Glutathione Peroxidase (GPX4)

**Fig. 7:**
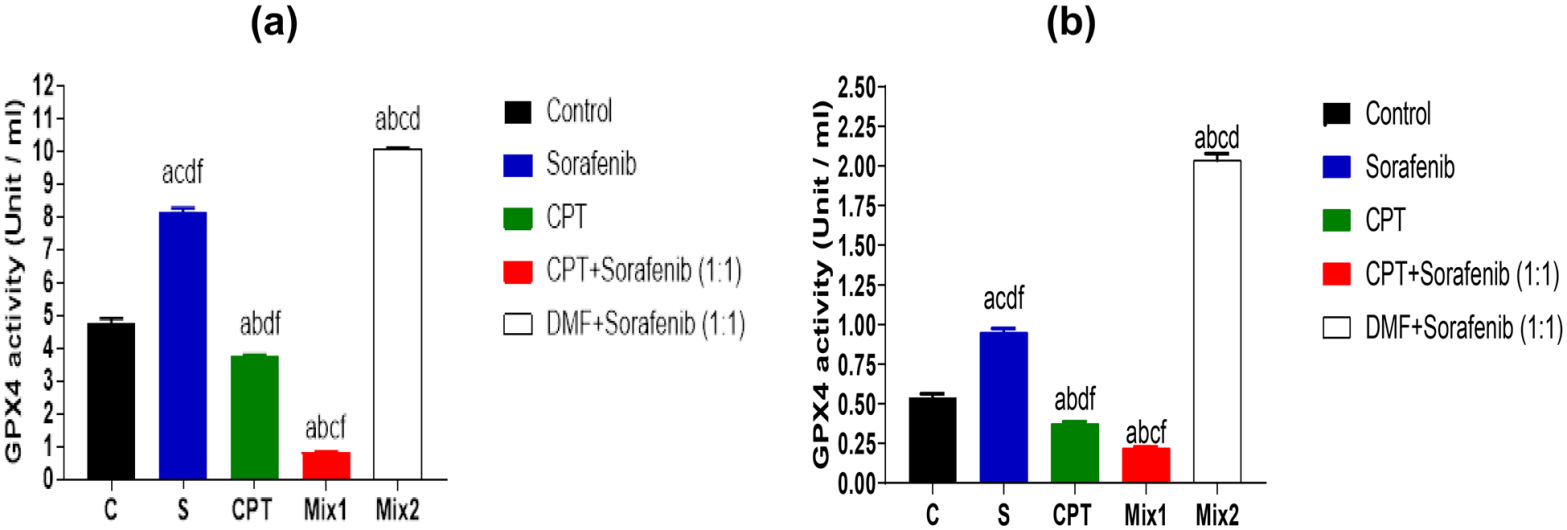
Glutathione Peroxidase (GPX4) activity in HepG2 **a** and Huh7 cells **b** after 72 hrs. of treatment with IC_50_ concentrations of sorafenib, CPT, mix 1 (combination of CPT and sorafenib), and mix 2 (sorafenib and Dimethyl fumarate). Data are presented as Mean ± SEM, n = 3/group. a: significant difference from control group, b: significant difference from sorafenib, c: significant difference from CPT group, d: significant difference from mix1 group, and f: significant difference from mix2 group, using one-way ANOVA followed by Tukey- Kramer multiple comparisons test.

In HepG2 cells, GPX4 activity showed a significant decrease in cells treated with mix 1 (0.8056 ± 0.02 U/ml) compared to both sorafenib (8.169 ± 0.1 U/ml*, p* < 0.0001), CPT (3.744 ± 0.04 U/ml, *p* < 0.0001), mix 2 (10.07 ± 0.03, *p* < 0.0001) and control groups (4.762 ± 0.15 U/ml, *p* < 0.0001) as shown in Fig. 7a. In addition, mix 2 showed statistically significant increase in GPX4 activity when compared to mix1, CPT, control and sorafenib (*p* < 0.0001, *p* < 0.0001,* p* < 0.0001,* p* < 0.0001, respectively).

Also, the same results occurred in Huh7 cell lysate, by using mix 1, the GPX4 activity decreased significantly (0.2196 ± 0.008 U/ml) compared to sorafenib (0.9517 ± 0.02 U/ml, *p* < 0.0001), CPT (0.3747 ± 0.01 U/ml, *p* < 0.01), mix 2 (2.036 ± 0.04 U/ml, *p* < 0.0001), and control groups (0.5412 ± 0.02 U/ml, *p* < 0.0001) as shown in Fig. 7b.

Moreover, mix 2 showed statistically significant increase in GPX4 activity when compared to mix 1, CPT, control and sorafenib (*p* < 0.0001, *p* < 0.0001,* p* < 0.0001, *p* < 0.0001, respectively).

These findings demonstrated that GPX4 activity in both cell line was declined after addition of CPT to sorafenib. So, sorafenib's ferroptosis activity may be improved by Nrf2 suppression caused by CPT.

#### Iron Concentration

**Fig. 8:**
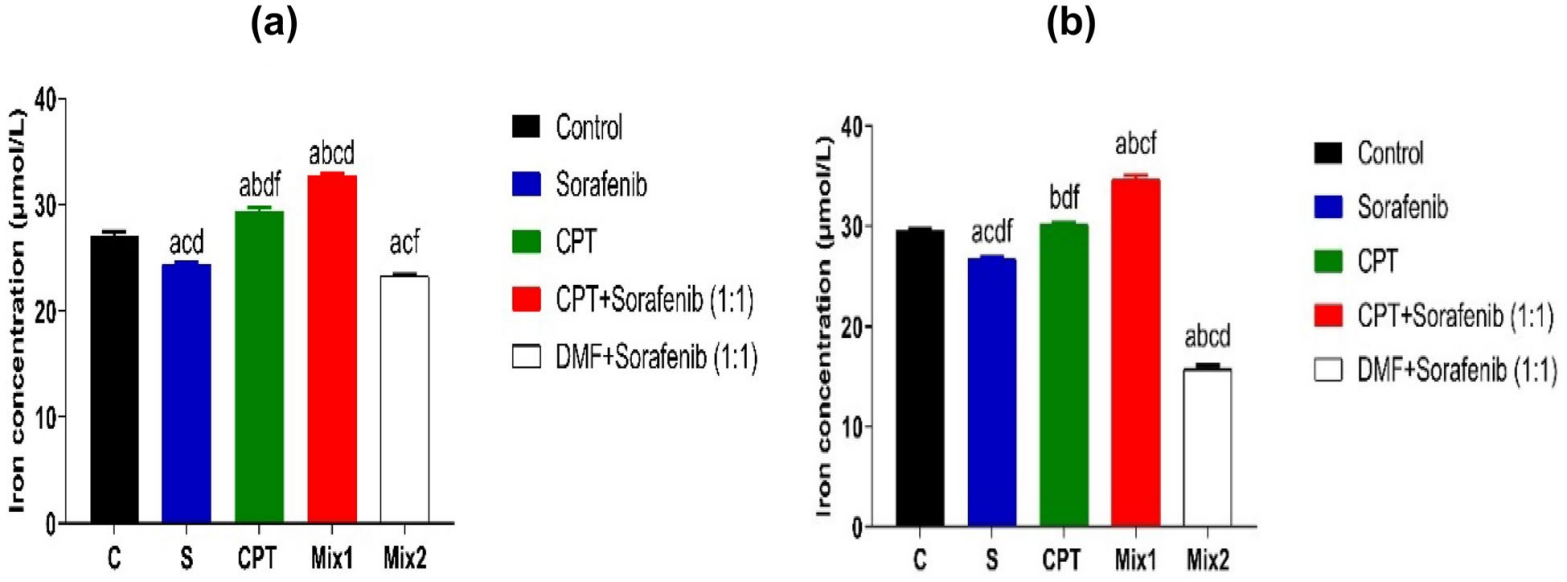
Iron concentration (µmol/L) in HepG2 **a** and Huh7 cells **b** after 72 hrs. of treatment with IC_50_ concentrations of sorafenib, CPT, mix 1 (combination of CPT and sorafenib), and mix 2 (sorafenib and Dimethyl fumarate). Data are presented as Mean ± SEM, n = 3/group. a: significant difference from control group, b: significant difference from sorafenib, c: significant difference from CPT group, d: significant difference from mix1 group, and f: significant difference from mix2 group, using one-way ANOVA followed by Tukey- Kramer multiple comparisons test.

In HepG2 cell line, mix 1 showed a significant increase in iron concentration (32.74 ± 0.2 µmol/L) compared to sorafenib (24.4 ± 0.14 µmol/L, *p* < 0.0001), CPT (29.39 ± 0.31 µmol/L,* p* < 0.0001) mix2 (23.24 ± 0.16 µmol /L,* p* < 0.0001) and control group (27.11 ± 0.39 µmol / L,* p* < 0.0001). Furthermore, the iron concentration was significantly decreased by using mix2 compared to control, mix1 and CPT (*p* < 0.0001, *p* < 0.0001, *p* < 0.0001, respectively) as shown in Fig. 8a.

While in Huh7 cell lysate, iron concentration increased significantly in the mix1 (34.6 ± 0.46 µmol/ml) compared to the sorafenib group (26.79 ± 0.12 µmol/L, *p* < 0.0001), CPT group (30.17 ± 0.2 µmol/L,* p* < 0.0001), mix2 (15.74 ± 0.4 µmol/L,* p* < 0.0001) and control group (29.64 ± 0.1 µmol/L,* p* < 0.0001) as shown in Fig. 8b. Additionally, mix 2 showed statistically significant decrease in iron concentration when compared to Control, sorafenib, mix1 and CPT (*p* < 0.0001, *p* < 0.0001, *p* < 0.0001, *p* < 0.0001, respectively). This may indicate that the combination of CPT with sorafenib causes an increase in iron concentration in both cell lines and accelerates cell death via ferroptosis.

### Effect of Sorafenib and CPT Combination on Protein Expression

For additional evaluation to the effect of a combination of CPT with sorafenib in reducing the resistance of sorafenib and improving the ferroptosis action on Huh7 and HepG2 cell lines, protein levels of Nrf2, ABCG2, MT-1G, p62 and SLC7A11 were determined using western blot analysis. The results showed that, in both cell lines, treatment with sorafenib alone made upregulation in the expression level of Nrf2, ABCG2, MT-1G and p62 proteins while made downregulation in the expression level of SLC7A11 protein compared to control untreated cells.

**Fig. 9:**
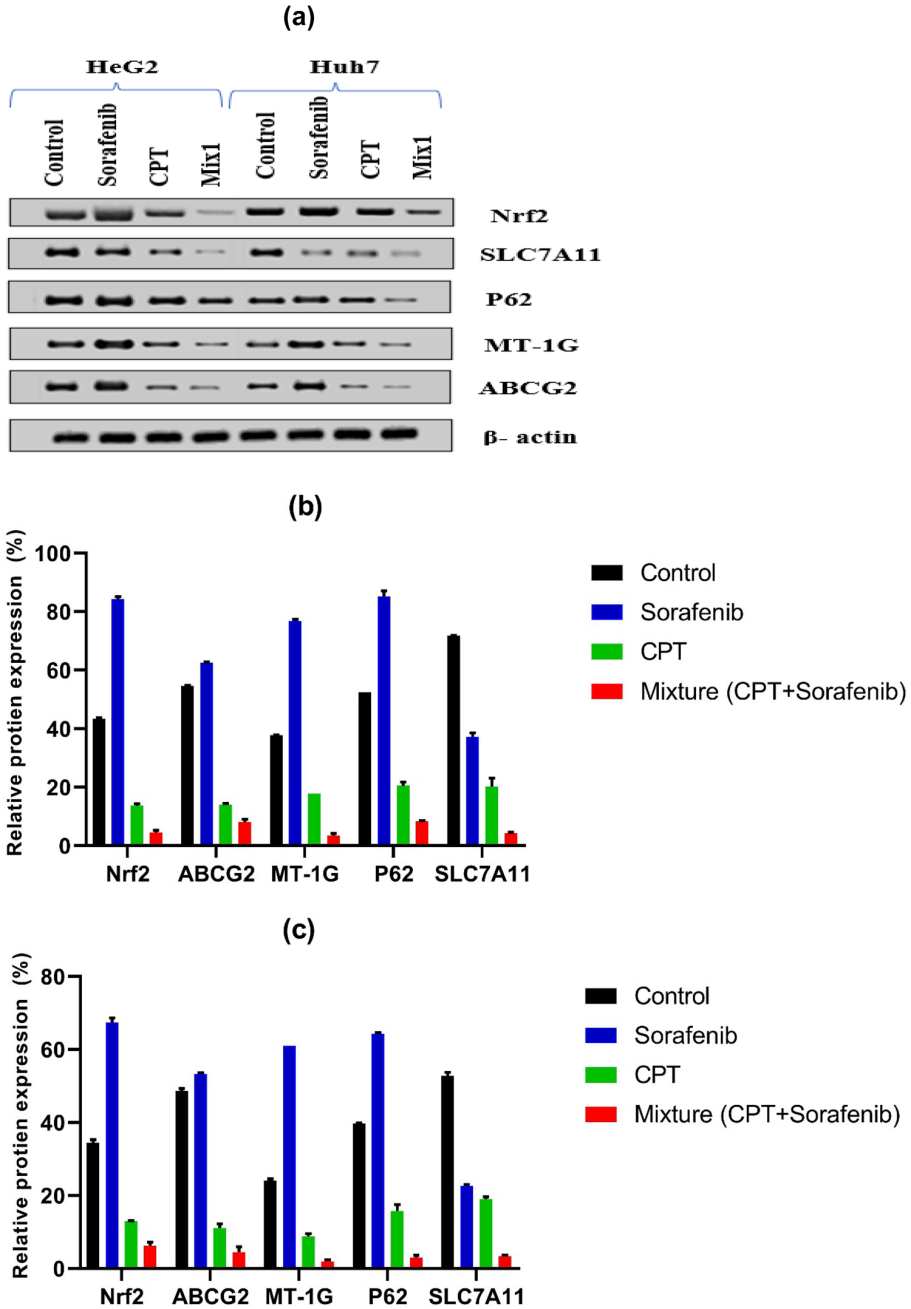
Protein expression (Western blot) analysis of Nrf2, ABCG2, MT-1G, p62 and SLC7A11 in Huh7 and HepG2 Cell lines. **a** The blots show the effect of IC_50_ concentrations of sorafenib, CPT and a combination of CPT and sorafenib at their reduced synergistic doses on the protein expression levels of Nrf2, ABCG2, MT-1G, p62 and SLC7A11 in Huh7 and HepG2 Cell lines treated for 72 hrs. The expression of the tested proteins was evaluated using one-way ANOVA followed by Tukey- Kramer multiple comparisons test in Huh7 **b** and HepG2 **c** Cell lysates and normalized to the β-actin protein. All groups in each protein are significant to each other. The data were shown as mean ± SEM.

Furthermore, combination of CPT and sorafenib at their lowered synergistic doses significantly decreased the expression levels of Nrf2, resistance proteins (ABCG2, MT-1G, p62) and ferroptosis transporter protein (SLC7A11) in both cell lines compared to the control, sorafenib and CPT groups as shown in Fig. 9a–c.

### Cellular Drug Uptake

To determine the effect of CPT on the uptake of sorafenib by HepG2 and Huh7 cells, the experiment was conducted on two separate plates one for sorafenib alone and the other for the mixture (sorafenib and CPT). The tests showed that the percentage of sorafenib uptake on HepG2 cells was significantly increased from 86.64% in cells treated with sorafenib alone to 98.95% (p < 0.01) in cells treated with combination of sorafenib and CPT.

**Fig. 10:**
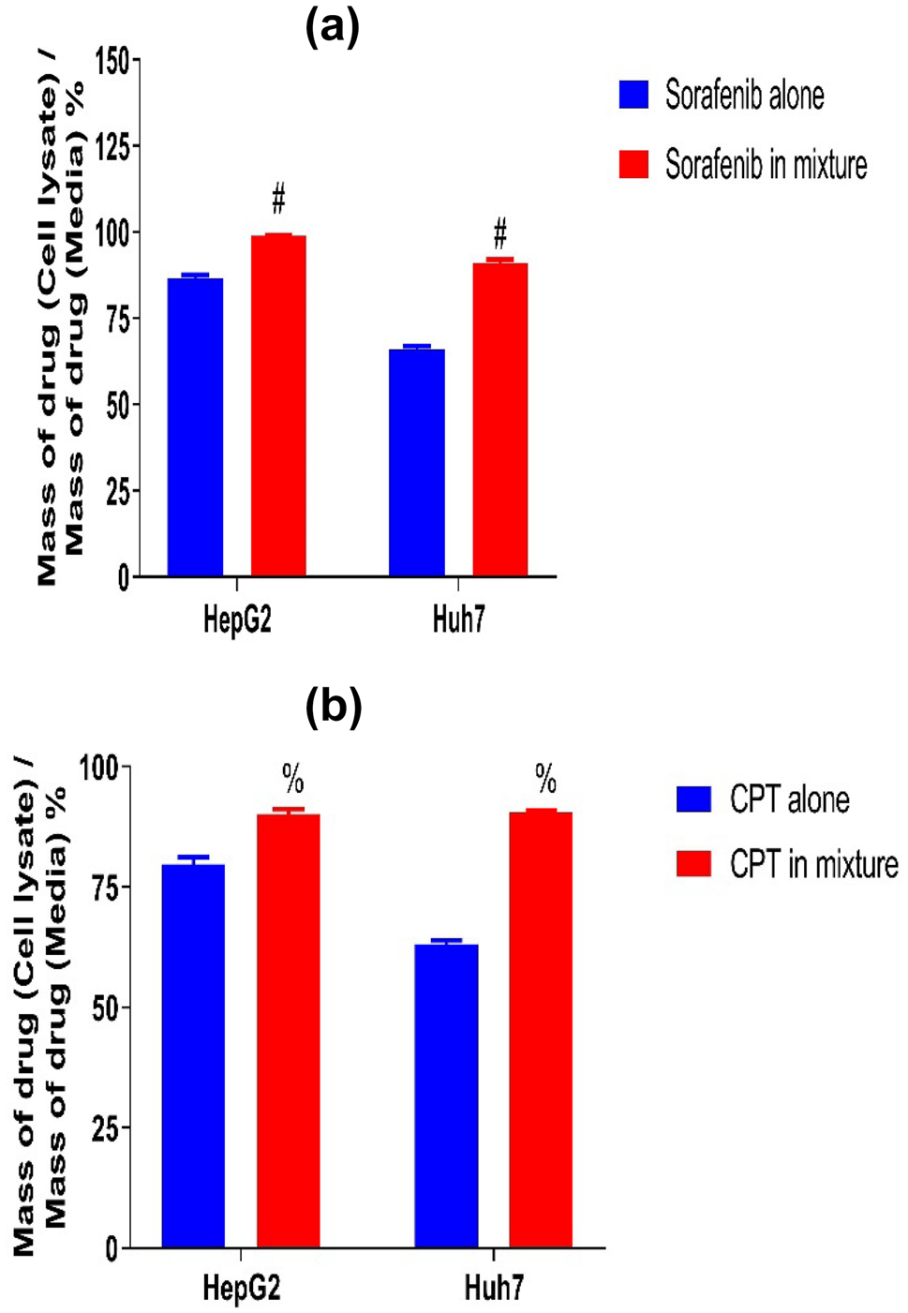
Percentage of sorafenib and CPT uptake in HepG2 and Huh7 cell lines determined by HPLC, **a** for sorafenib and **b** for CPT. Drug amount in cells compared to initial drug amount in media was used to calculate the data.; the values represent the percentage of drugs taken into cells. The data are shown as mean ± SEM using unpaired student t- test. #: significant difference from sorafenib alone, %: significant difference from CPT alone.

While the uptake of sorafenib by Huh7 cells was significantly increased from 65.94% in cells treated with sorafenib alone to 91.03% (*p* < 0.001) in cells treated with a combination of sorafenib and CPT as shown in Fig. 10a.

Furthermore, the uptake of CPT was significantly increased from 79.6% to 90.1% (*p* < 0.01) and from 63.3% to 90.5% (p < 0.001) in HepG2 and Huh7 cell line, respectively Fig. 10b. These results indicated that cellular accumulation of both drugs increased in case of combination compared to each drug alone.

### Bioinformatics Analysis of Correlation Between Proteins

**Fig. 11:**
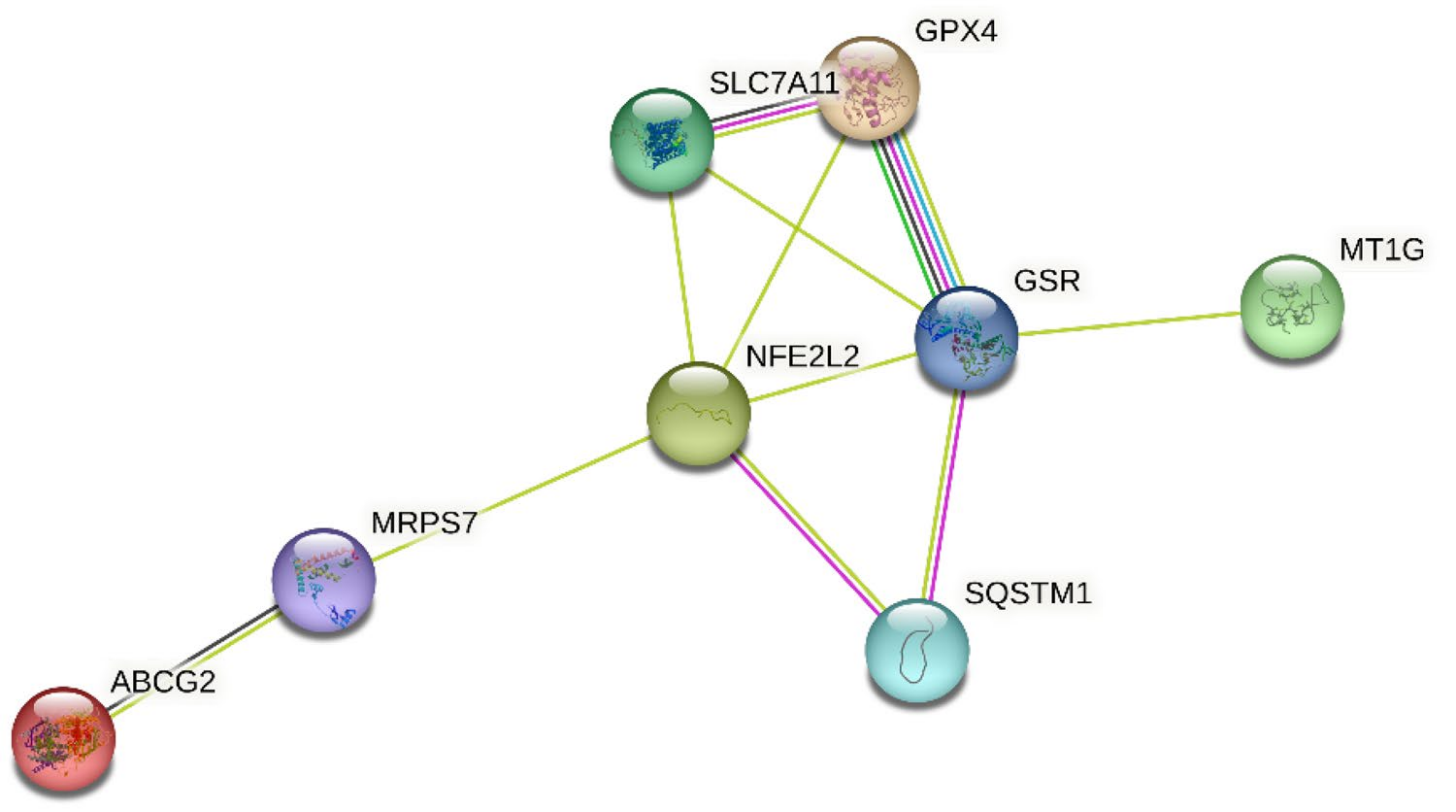
Protein -protein interaction network between Nrf2, ABCG2, p62, MT-1G, GPX4, GR and SLC7A11 proteins.

The results of string website found protein -protein correlation among Nrf2, resistance proteins (p62, MT-1G and ABCG2) and ferroptosis proteins (GR, GPX4 and SlC7A11) as shown in Fig. 11.

## DISCUSSION

In the past few decades, although a number of studies have been carried out to improve the efficacy of sorafenib by overcoming its resistance and reducing its side effects [31], it is still a major clinical challenge to use sorafenib in the treatment of advanced HCC. The Nrf2 activation by sorafenib induces tumor development [16], suppresses sorafenib’s ferroptosis action [32], potentiates sorafenib’s resistance [33], and decreases sorafenib’s uptake [14]. Consequently, our study tried to shed light on a new combination of sorafenib with CPT (Nrf2 inhibitor) to investigate their effects on ferroptosis and several resistance pathways implicated in HCC using HepG2 and Huh7 cell lines.

Our study demonstrated that cell viability, CI and DRI indicated strong synergism between sorafenib, and CPT. CPT potentiates sorafenib efficacy by reducing its IC_50_ in HepG2 and Huh7 cell lines compared to IC_50_ of sorafenib alone after 48 and 72 hrs. of incubation. This results strongly recommended that down-regulation of Nrf2 by CPT favored the inhibition of HCC growth by sorafenib.

It is well evidenced that p62-Keap1-Nrf2 axis is one of the most critical pathways implicated in HCC carcinogenesis [34]. Also, the over expression of Nrf2 induces cell proliferation in HCC by reducing ferroptosis [35], redirecting glucose and glutamine into anabolic pathways as the de novo nucleotide synthesis and the NADPH production [36]. These findings were matched with many studies which suggested that using pharmacological Nrf2 inhibitors may improve chemotherapeutic sensitivity like metformin enhanced sorafenib efficiency in HCC [20] and ML385 drug also enhanced sorafenib sensitivity in lung cancer [37]. Sorafenib could kill the tumor by inducing ROS accumulation and promoting the overload of lipid peroxide [6].However, the high ROS level induced by sorafenib may result in abnormal activation of the p62–Keap1–Nrf2 pathway [10].

Abnormal activation of Nrf2 enhances transcription of anti-ferroptosis downstream genes as solute carrier family 7 membrane 11 (SLC7A11/xCT), NAD(P)H quinone oxidoreductase 1 and detoxifying enzymes (e.g., GSH S-transferase, UDP-glucuronosyltransferase, GPX4, GSH reductase), reducing the sensitivity of ferroptosis in the end [11]. CPT acts by suppressing SCL7A11 [38] and Nrf2 [3, 17] but the role of CPT in improving sorafenib’s ferroptosis action is still unclear.

In our study, CPT inhibited Nrf2 and SLC7A11 expression effectively compared to control (Untreated group). While sorafenib could increase ROS level and induce high Nrf2 expression compared to control. Additionally, we found that the combination of sorafenib and CPT significantly increased lipid peroxidation by (218.8% and 152.1%), iron concentration by (134%, 129%), decreased TAC by (54.1% and 68.08%), decreased GR (63.4% and 57.9%), decreased GPX4 activity (10%, 23%) and reduced the expression of SLC7A11 compared to sorafenib alone in HepG2 and Huh7, respectively.

For more confirmation, Nrf2 activator compound DMF was used [23]. It was found that the combination of sorafenib and DMF increased both TAC and iron concentration, while decreased both (GR and GPX4 activity) which finally led to a decrease in ferroptosis action of sorafenib in HCC cells. Our results were consistent with several studies showing that Nrf2 inhibition is considered an option to improve the action of ferroptosis inducer [33, 39–41].

CPT alone or sorafenib alone can activate autophagy to maintain cell survival by decreasing the ROS (ferroptosis) and reducing the drug’s apoptosis action [42, 43]. Besides, activating the p62 –Keap1-Nrf2 pathway [44], and MT-1G reduce ROS and induces autophagy for cellular homeostasis [45]. But our results showed that, a combination of sorafenib and CPT induced cell death through increasing ROS and decreasing expression of (p62, MT-1G and Nrf2). These results suggest that this combination may not activate autophagy.

In addition, CPT alone or sorafenib alone can also activate the apoptosis mechanism to induce cell death [46, 47]. Induction of Nrf2 prevents the expression of apoptotic marker like Bcl-xL[48].

By addition of ZVAD-FMK (an apoptosis inhibitor) to the combination of sorafenib and CPT, there was no change in cell viability compared to cells treated with a combination of sorafenib and CPT alone. So, our results showed that combination treatment did not suppress HCC cells proliferation through apoptosis. Moreover, the concentration of the combination used in our experiment is considerably lower than the concentration of sorafenib and CPT that other researchers have shown is necessary to induce apoptosis in HCC cell lines [46–50].

On the other hand, cells treated with ferrostatin-1 (a ferroptosis inhibitor) to the combination of sorafenib and CPT, showed a significant increase in cell viability compared to our combination (sorafenib and CPT alone). These findings indicate that the combination of sorafenib and CPT suppressed HCC cell proliferation via ferroptosis.

Nrf2 supposedly induces sorafenib’s resistance through controlling the transcriptional production of some resistance genes such as Metallothionein-1G (MT-1G) [13] and p62 [12]. Sun et al. demonstrated that after ferroptosis activation by sorafenib, Nrf2 induces expression of MT-1G that meditated sorafenib resistance by inhibiting its ferroptosis action, at which MT-1G inhibits lipid peroxidation and induces GSH production [34]. Also, Pan et al. revealed that after sorafenib induction of ferroptosis, Nrf2 induces the p62 expression. At the same time, p62 contributes to activate the Nrf2 by autophagic degradation of Keap1.This activation of Nrf2 mediates sorafenib resistance by inhibiting its ferroptosis action [12].

In our study, Nrf2 inhibition by CPT reduces the expression of MT-1G and p62 compared to sorafenib alone. This is the first research that has been done, as far as we are aware, to examine the role of CPT in lowering sorafenib resistance markers.

These findings were similar to those in several studies suggesting that using Nrf2 pharmacologic suppressor reduces chemotherapeutic resistance [40, 41, 51]. In addition, our work showed that CPT effectively reduced Nrf2 expression at μ M concentration lower than other Nrf2 inhibitors concentration [3, 17].

Furthermore, sorafenib’s resistance can occur by overexpression of drug efflux transporters, such as ATP-binding cassette (ABC), which is upregulated by Nrf2 [52]. Several studies demonstrated that ABCG2 is a major transporter for the efflux of sorafenib [14, 53] and sorafenib-resistant HCC cells also expressed higher levels of ABCG2 [54] but till now there is no study explaining the effect of inhibition Nrf2 on reduction sorafenib’s efflux and resistance. Our findings showed for the first time that combination of CPT and sorafenib downregulate ABCG2 expression compared to sorafenib alone and consequently, the sorafenib intracellular accumulation was increased from 86.64% to 98.95% in HepG2 and from 65.94% to 91.03% in Huh7 cells. We can therefore conclude that ABCG2 induces sorafenib resistance by inhibiting its ferroptosis action via promoting its efflux. For more confirmation, STRING bioinformatics tool was used. The results found protein -protein correlation between Nrf2, resistance proteins (p62, MT-1G and ABCG2) and ferroptosis proteins (GR, GPX4 and SLC7A11). Interestingly, we also found that the combination lowers the CPT's efflux. Additional study is required to fully understand this finding's mechanism.

**Fig. 12:**
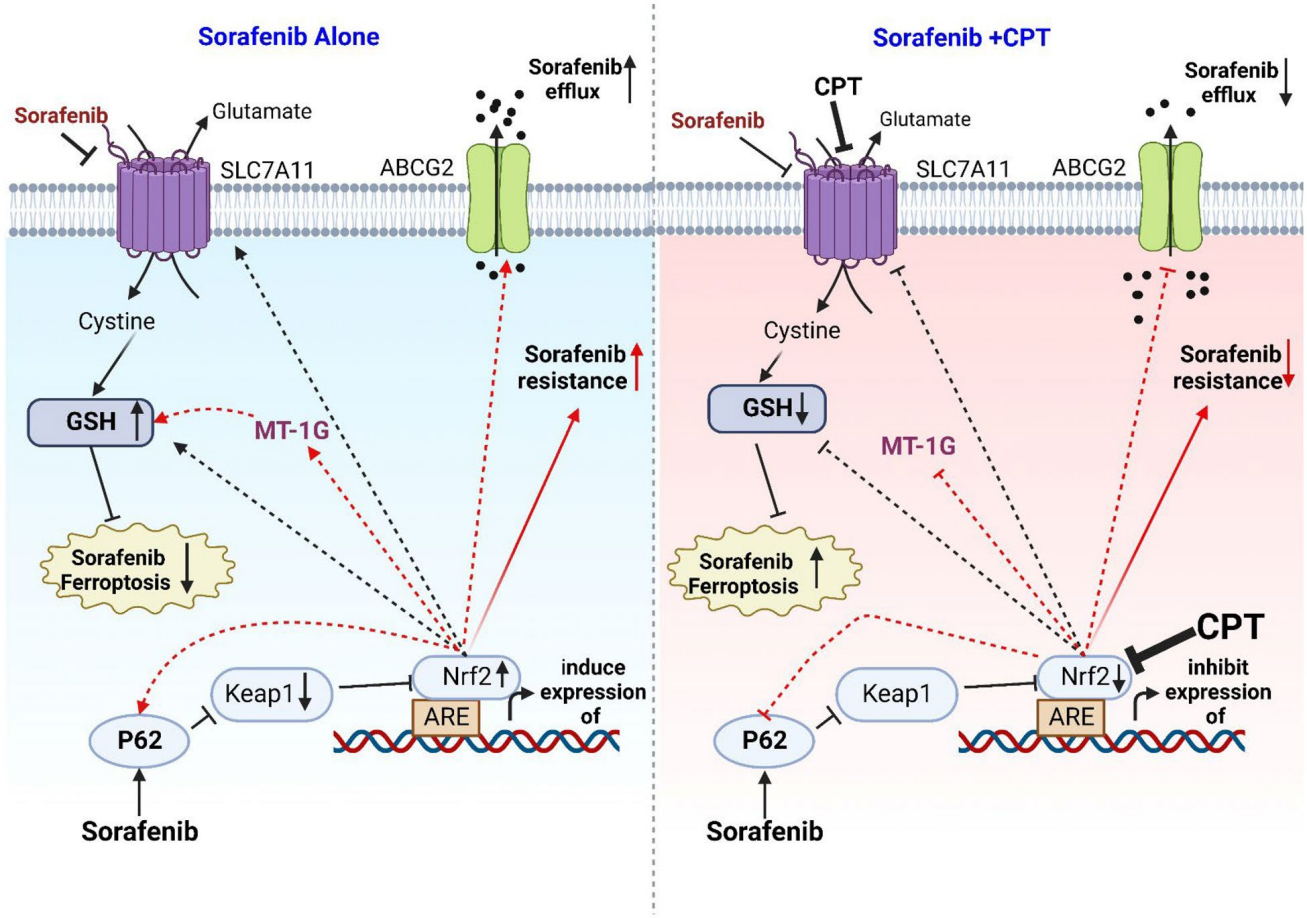
Graphic abstract showed that sorafenib alone induces the ferroptosis but at same time induces p62-Keap1-Nrf2 pathway. The activation leads to induce expression of SLC7A11, GSH, p62, MT-1G and ABCG2 proteins. After treatment of HCC cell by CPT with Sorafenib, all proteins were downregulated. Therefore, sorafenib’s sensitivity and ferroptosis action were improved while sorafenib’s resistance and efflux were reduced.

The present investigation may provide evidence on a potent synergism between sorafenib and CPT in the treatment of HCC. Nrf2 inhibition by CPT improves sorafenib’s efficacy and reduces its resistance through augmenting sorafenib’s ferroptosis action. Furthermore, down regulation of ABCG2 by this combination induces sorafenib’s uptake by HCC cells. The following graphical abstract illustrates our study's concluding remarks Fig. 12.

## Data Availability

All data generated or analyzed during this study are included in this published article.
